# Glycemic control after switching to faster aspart in adults with type 1 diabetes

**DOI:** 10.1007/s40618-022-01745-2

**Published:** 2022-02-01

**Authors:** G. P. Fadini, F. Boscari, D. Falaguasta, S. Ferretto, A. Maran, A. Avogaro, D. Bruttomesso

**Affiliations:** 1 Department of Medicine - DIMED, Division of Metabolic Disease, University of Padova, Via Giustiniani 2, 35128 Padua, Italy; 2 Division of Metabolic Disease, University Hospital of Padova, Padua, Italy

**Keywords:** Post-prandial hyperglycemia, Time in range, Observational, Bolus insulin

## Abstract

**Aims:**

Post-prandial hyperglycemia remains an unmet need in the management of type 1 diabetes (T1D). In randomized trials, faster insulin aspart (FIA) showed modest but significant reductions of glycemic spikes after meals. Whether such benefit is evident in routine clinical practice is unclear.

**Methods:**

We analyzed data of patients with T1D at the time they switched from a prior bolus insulin to FIA and at the first available follow-up. The primary endpoint was the change in the time spent in hyperglycemia > 250 mg/dl during daytime from flash glucose monitoring (FGM). Secondary outcomes included the change in HbA1c, body weight, insulin dose and other FGM metrics.

**Results:**

We included 117 patients with T1D on multiple daily injections who switched to FIA, 57 of whom had data from FGM. Patients were 41-year-old, 51.3% men, with 19.3 years diabetes duration and a baseline HbA1c of 7.7% (60 mmol/mol). Mean observation time was 4.3 months. After switching to FIA, HbA1c declined by 0.1% (1 mmol/mol) only in patients with baseline HbA1c > 7.0% (53 mmol/mol). Time spent in hyperglycemia > 250 mg/dl during daytime was significantly reduced from 14.8 to 11.9% (*p* = 0.006). Time in range improved from 48.3 to 51.0% (*p* = 0.028). Results were consistent across various patient characteristics.

**Conclusions:**

Under routine care, patients with T1D who switched to FIA experienced a reduction in the time spent in hyperglycemia > 250 mg/dl during daytime and an increase in time in range. These improvements may be due to better control of post-prandial hyperglycemia, as observed in trials.

## Introduction

In people with type 1 diabetes (T1D), post-prandial glucose excursions contribute to increasing glucose variability and decreasing time in range (TIR). This is demonstrated by studies on a dedicated algorithm to control post-prandial glucose with closed loop insulin delivery (1) and by studies showing post-prandial glucose control with a sodium-glucose co-transporter inhibitor (2).

Early (1 h) post-prandial hyperglycemic spikes emerge as better determinant of metabolic dysregulation and vascular alterations than later (2 h) glycemic values (3). Post-prandial hyperglycemia also worsens quality of life and is associated with loss of productivity (4–6). Therefore, mitigating post-prandial hyperglycemia is one of the aims of T1D glycemic management. To this end, interventions that approximate physiological post-prandial responses should actively be sought to reduce the risk of complications (7). Despite decades of optimization of basal-bolus insulin therapy, handling of post-prandial hyperglycemia with insulin injections remains an unmet need (8).

Faster insulin aspart (FIA) is a modified formulation of aspart with excipients that allow faster absorption of insulin monomers from the subcutaneous depot (9). The resulting anticipated T_max50%_ and increased early exposure (AUC_0–30 min_) grant a greater glucose-lowering effect within 30 min after injection (10). In a meta-analysis of randomized controlled trials (RCTs), use of FIA as compared to insulin aspart among individuals with T1D achieved a modest but significant reduction in HbA1c ( – 0.08%) and a reduction in 1 h glucose excursion after a meal test ( – 0.9 mmol/L; 16 mg/dl) (11). These effects are expected to improve TIR, defined as the time a patient spends in a given glycemic interval (usually 70–180 mg/dl). TIR is emerging as a novel metric of glucose control beyond HbA1c (12). Due to hyper- and hypoglycemic excursions, TIR can vary substantially for any given HbA1c value, implying that HbA1c can be misleading when used as the sole metric to judge glucose control (13). In fact, in people with T1D, TIR is associated with the risk of microvascular complications and provides incremental value when added to HbA1c to predict complications (14).

The widespread availability of flash glucose monitoring (FGM) among people with T1D allows an unprecedented opportunity to demonstrate early post-prandial hyperglycemia and assess TIR in routine clinical practice (15, 16). This also enables the real-world evaluation of new therapies, like FIA, that are expected to blunt glycemic excursions. To date, the published real-world experience with FIA in T1D is limited to results of the for-profit, multicenter, single-arm, non-interventional GoBolus study (17). Among 243 middle-aged (50 year) patients with T1D since about 19 years and a baseline HbA1c of 8.1% (65 mmol/mol), TIR improved from 46.9 to 50.1%, due to a reduction in the time spent in hyperglycemia and no change in the time spent in hypoglycemia.

In the present study, we wished to confirm RCT findings and prior real-world experience on the effectiveness of FIA in reducing hyperglycemia and improving TIR. To this end, we performed a retrospective cohort study under free-living conditions, by collecting clinical data and, when available, FGM data from patients with T1D who switched from other bolus insulins to FIA.

## Methods

### Study design and data source

This was a retrospective, observational study with longitudinal data collection. The study was conducted in agreement with the principles of the declaration of Helsinki. In accordance to national regulations on retrospective studies, the protocol was cleared to by the Ethical Committee of the University Hospital of Padova (prot. no. 177n/AO/21). Patients provided written informed consent for the re-use of routinely collected clinical data for research purposes.

The source of data for this study was the electronic chart system of the diabetes outpatient clinic of the University Hospital of Padova, which stores information on all consecutive visits of all patients attending the clinic, including demographics, anthropometrics, lab exams, complications, and therapies. The data collection period was from April 2018 to March 2020.

### Patients

Inclusion criteria were as follows: a diagnosis of T1D since at least one year; continuous use of basal-bolus insulin since at least 1 year; a switch from aspart, lispro or glulisine to FIA; availability of at least one follow-up examination after a minimum of 2 months and a maximum of 9 months since index date; still being on FIA at follow-up. The index date was set as the date patients initiated FIA. Date of the follow-up examination was set as the date patients re-accessed the clinic (2–9 months after index date) still being on FIA. We retrospectively ensured that no substantial change in dietary and exercise habits was requested from these patients. Patients were excluded if they were on continuous subcutaneous insulin infusion, or stopped FIA before returning to the clinic, or had missing data for evaluation of the outcomes.

Patients using FGM were a subgroup of the total population of included participants. To be included in this subgroup, patients needed to have complete data for 4 weeks before index data and 4 weeks before follow-up date, with sensor coverage of 90% or greater.

### Data collection

We collected the following data for all patients: demographics (age, sex); diabetes duration; anthropometrics (height and weight for the calculation of BMI); data on concomitant risk factors (obesity, hypertension, dyslipidemia) and thyroid comorbidity; standard metrics of glucose control (HbA1c and fasting plasma glucose); complications (defined as coded in the electronic chart system, as described before (18)) with particular reference of micro- (retinopathy, nephropathy, neuropathy) and macroangiopathy (coronary, cerebral or peripheral); type and doses of previous bolus insulin; type and doses of ongoing basal insulin; other medications. Baseline data were collected closest to the index date (< 3 months for laboratory data and < 6 months for comorbidities).

For patients included in the FGM subgroup, we collected all FGM values at 15-min intervals from the cloud system (Libreview) connected to patients’ smartphone applications. We calculated the following metrics from the 4 weeks preceding index date and the 4 weeks preceding follow-up date: average glucose, standard deviation, coefficient of variation, time in range (70–180 mg/dl), time spent in hypoglycemia 54–70 mg and < 54 mg/dl; time spent in hyperglycemia 180–250 mg/dl and > 250 mg/dl. All metrics were re-calculated from raw data for day-and-night together and for the daytime (6am to 12 pm) and nighttime (0–6am) separately.

### Outcome variables

We collected updated values for the following outcome variables for all patients, closest to the follow-up date but after index date: HbA1c, fasting glucose, body weight, bolus and basal insulin dose. In the entire study cohort, outcome measures were the change in HbA1c, fasting glucose, weight and insulin doses. For patients in the FGM group, the primary endpoint was the change in the time spent in hyperglycemia > 250 mg/dl, which is expected to best reflect hyperglycemic spikes occurring after meals and be ameliorated by FIA. All other sensor metrics were secondary outcomes.

### Statistical analysis

Continuous variables are presented as mean (standard deviation), whereas categorical variables are presented as percentage. The change from baseline in continuous variables were assessed using paired Student’s *t* test. To adjust for different FIA exposure time (from baseline to follow-up), we used a generalized linear model for repeated measures. Statistical significance was accepted as *p* < 0.05 and SPSS ver. 23 was used.

## Results

### Patient characteristics

A total of 191 patients initiating FIA between April 2018 and March 2020 were initially screened for available data. We excluded patients with type 2 diabetes, those using an insulin pump or who did not have valid follow-up data, and those who discontinued FIA. Thus, we finally included in the analysis 117 patients with T1D (51.3% men) who were, on average, 40.9 years old and had a diabetes duration of 19.4 years (Table 1). Baseline HbA1c was 7.7% (60 mmol/mol), 48.7% had at least one microvascular complication, while macrovascular complications were much rarer (6.8%). As for the ongoing therapy at the time of switch, a similar number of patients were on insulin aspart (42.7%) and lispro (42.6%,), while much less were on glulisine (15.4%). Long-acting insulin were distributed as follows: 27.4% were on glargine-100; 24.8% were on glargine-300; 47.9% were on degludec. A minority of patients were also on metformin or an SGLT-2 inhibitor. Fifty-seven patients were using a FGM and their characteristics are reported in Table 1.

**Table 1:** Clinical characteristics

	All patients	SMBG	FGM
Demographics	*N* = 117	*N* = 60	*N* = 57
Age, years	40.9 (14.6)	44.7 (15.1)	36.9 (13.0)
Sex male, %	51.3	48.3	54.4
Diabetes duration, years	19.4 (11.8)	21.2 (11.5)	17.3 (11.6)
*Risk factors and comorbidities*			
Obesity, %	6.0	8.3	3.5
BMI, kg/m^2^	24.2 (3.6)	24.4 (3.8)	24.1 (3.5)
Body weight, kg	71.2 (13.7)	71.0 (14.6)	71.5 (12.9)
Hypertension, %	34.2	41.7	26.3
Systolic blood pressure, mm Hg	131.4 (16.1)	133.7 (18.9)	128.9 (12.1)
Diastolic blood pressure, mm Hg	78.1 (9.4)	78.0 (10.8)	78.2 (7.7)
Dyslipidemia, %	35.0	33.3	36.8
Total cholesterol, mg/dl	177.4 (29.4)	173.8 (25.7)	181.7 (32.9)
HDL cholesterol, mg/dl	62.6 (15.1)	62.0 (14.3)	63.4 (16.0)
Triglycerides, mg/dl	81.5 (41.3)	83.4 (40.3)	79.3 (42.8)
LDL cholesterol, mg/dl	98.5 (26.6)	95.1 (24.7)	102.4 (28.3)
Thyroid disease, %	14.5	13.3	15.8
Glucose control			
Fasting plasma glucose, mg/dl	163.6 (67.1)	161.1 (60.5)	166.8 (76.0)
HbA1c, %	7.7 (0.8)	7.7 (0.8)	7.7 (0.8)
*Complications*			
Cardiovascular disease, %	1.7	3.3	0.0
Chronic kidney disease, %	3.2	5.7	0.0
eGFR, ml/min/1.73 m^2^	102.0 (20.1)	98.9 (21.8)	105.9 (17.2)
Micro-/macro-albuminuria, %	6.0	3.3	8.8
Retinopathy, %	38.0	41.0	34.4
Neuropathy, %	41.8	48.3	34.6
Any macroangiopathy, %	6.8	10.0	3.5
Any microangiopathy, %	48.7	53.1	43.9
*Previous bolus insulin*			
Aspart, %	42.7	46.7	40.4
Lispro, %	43.6	45.0	38.5
Glulisine, %	15.4	8.3	22.8
*Basal insulin*			
Glargine-100, %	27.4	21.7	33.3
Glargine-300, %	24.8	22.3	26.3
Degludec-100, %	47.9	55.0	40.4
*Other drugs*			
Metformin, %	7.7	8.3	7.0
SGLT2i, %	3.4	3.3	3.5
RAS blockers, %	10.3	10.0	10.5
Statin, %	17.1	21.7	12.3
Anti-platelet agents	1.7	1.7	1.8

*BMI* body mass index, *HDL* high density cholesterol, *LDL* low density cholesterol, *eGFR* estimated glomerular filtration rate, *SGLT2i* sodium-glucose co-transporter-2 inhibitors, *RAS* renin angiotensin system. Data are presented as mean (standard deviation) or as percentage

### Change in clinical-laboratory parameters

In the entire cohort, patients were re-assessed a median of 4.2 months (IQR 3.4–5.3) after initiation of FIA (index date). We observed no change in HbA1c, fasting glucose or body weight. While bolus insulin doses remained unchanged, basal insulin dose slightly but significantly increased by an average 0.3 IU (Table 2). The change in HbA1c was statistically significant only among patients with a baseline HbA1c above 7.0% (53 mmol/mol):  – 0.09; 95% CI  – 0.004% to  – 0.18% (equal to  – 0.9 mmol/mol; 95% CI  – 0.04 to  – 1.8).

**Table 2:** Change in clinical parameters

Variable	Baseline	Follow-up	*p* value
HbA1c, %	7.7 (0.8)	7.6 (0.8)	0.300
Fasting glucose, mg/dl	163.6 (67.1)	168.4 (72.1)	0.474
Body weight, kg	71.2 (13.6)	71.2 (13.8)	0.788
Basal insulin (mean daily dose), IU/kg	0.27 (0.11)	0.28 (0.10)	0.023
Basal insulin (mean daily dose), IU	19.3 (8.4)	19.6 (8.3)	0.029
Bolus insulin (mean daily dose), IU/kg	0.33 (0.15)	0.32 (0.15)	0.783
Bolus insulin (mean daily dose), IU	23.0 (10.9)	23.0 (11.4)	0.949

These endpoints have been analyzed in the entire population of 117 patients. Data are presented as mean (standard deviation)

### Change in sensor metrics

In the subgroup of patients using FGM (all with first-generation sensors), the median follow-up time was 4.1 months (IQR 3.4–5.0). The change in HbA1c in this subgroup of patients was similar to that observed in the entire population, as the effect was significant only for patients with baseline HbA1c > 7.0% (53 mmol/mol):  – 0.09; 95% CI  – 0.0001% to  – 0.18% (equal to  – 0.9 mmol/mol; 95% 0–01 to  – 1.8).

**Fig. 1:**
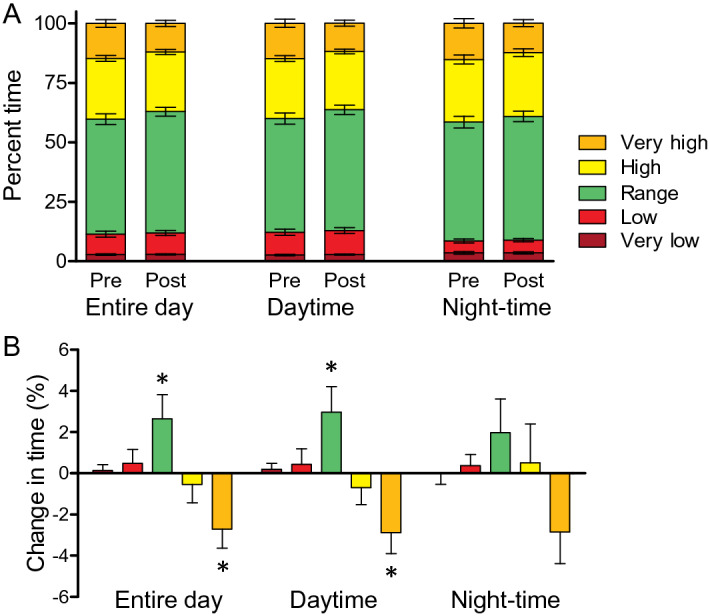
Change in sensor metrics. **A** Time spent in the various glucose ranges before (pre) and after (post) switching to FIA. The metrics are calculated in the entire day, daytime and nighttime separately. **B** Changes in time spent in the various glucose range (same legend as in panel **A**). *p < 0.05

Table 3 and Fig. 1A show the change in glucose sensor metrics We observed a significant reduction in average glucose levels during the entire time of the day, which was mostly attributable to a reduction in average glucose during daytime (from mean ± SD 162.2 ± 34.7 to 155.0 ± 27.2; *p* = 0.016). Nighttime improvement in average glucose was smaller and not statistically significant. The time spent in hyperglycemia > 250 mg/dl during day-and-night decreased significantly, which was attributable to a reduction during daytime (from 14.8 ± 12.8%% to 11.9 ± 9.7%; *p* = 0.006). This finding was confirmed after adjusting for FIA exposure time (from 15.4% to 11.3%; *p* < 0.001).

**Table 3:** Metrics from the flash glucose monitoring system

	Day and night			Nightime (0:00–6:00am)			Daytime (6:00am – 0:00)		
	Baseline	Follow-up	*p* value	Baseline	Follow-up	*p* value	Baseline	Follow-up	*p* value
Mean, mg/dl	163.7 (33.8)	157.0 (26.1)	0.021	169.7 (39.0)	164.1 (30.8)	0.131	162.2 (34.7)	155.0 (27.2)	0.016
SD, mg/dl	76.6 (21.1)	75.1 (19.7)	0.243	69.3 (20.8)	67.6 (20.6)	0.504	77.5 (22.6)	75.8 (20.6)	0.198
CV, %	48.0 (14.1)	48.5 (12.6)	0.676	42.1 (13.2)	41.3 (10.7)	0.594	49.1 (15.4)	49.9 (14.2)	0.540
Very low, % < 54 mg/dl	2.8 (2.5)	2.9 (1.9)	0.621	3.5 (3.8)	3.5 (3.1)	0.985	2.6 (2.5)	2.8 (2.0)	0.517
Low, % 54–69 mg/dl	8.6 (8.7)	9.0 (8.2)	0.482	5.0 (6.0)	5.4 (5.4)	0.491	9.6 (9.8)	10.1 (9.5)	0.571
Range, % 70–180 mg/dl	48.3 (17.0)	51.0 (14.4)	0.028	50.0 (18.5)	52.0 (16.9)	0.234	47.8 (17.6)	50.8 (14.7)	0.020
High, % 181–250 mg/dl	25.6 (9.4)	25.1 (7.7)	0.544	26.3 (14.9)	26.8 (12.2)	0.789	25.2 (9.0)	24.5 (7.5)	0.402
Very high, % > 250 mg/dl	14.7 (12.1)	12.0 (9.5)	0.005	15.2 (15.2)	12.4 (11.4)	0.070	14.8 (12.8)	11.9 (9.7)	0.006

Data are presented as mean (standard deviation). *SD* standard deviation, *CV* coefficient of variation

The improvement in nighttime spent in hyperglycemia > 250 mg/dl was quantitatively similar but not statistically significant (*p* = 0.07) due to greater variability (SD) of the effect. Time spent in hypoglycemia did not change. An increase in time in range was observed, which was significant during daytime (from 47.8 ± 17.6% to 50.8 ± 14.7; *p* = 0.020) but not during nighttime (Fig. 1B). Metrics of glucose variability (standard deviation and coefficient of variation) remained unchanged.

**Fig. 2:**
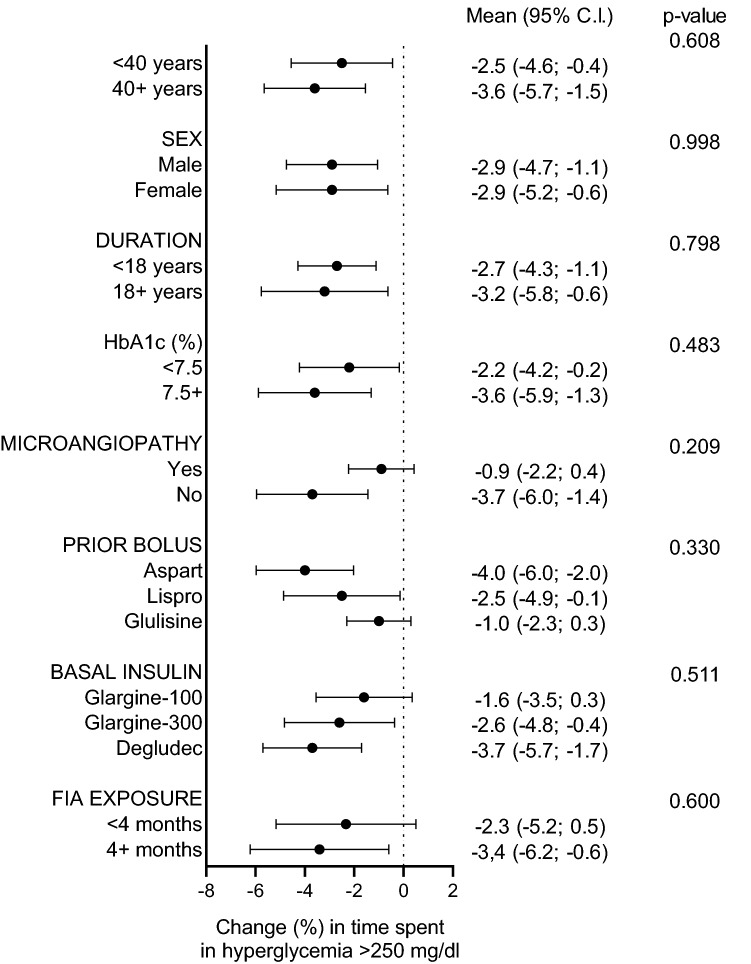
Subgroup analysis. Change in the percentage of time spent in very high glucose during daytime according to patient characteristics and duration of exposure. Data are presented as mean (95% CI). *P* values are reported for the comparison between subgroups defined by clinical variables

We then stratified patients according to key clinical characteristics, including age, sex, diabetes duration, baseline HbA1c, presence/absence of microangiopathy and type of prior insulin therapy. The change in time spent in hyperglycemia > 250 mg/dl was consistent in subgroups and not significantly modified by any of these baseline clinical characteristic or duration of exposure (Fig. 2).

## Discussion

In this study, we show that patients with T1D on multiple daily insulin injections and suboptimal glycemic control who switched from other bolus insulins to FIA experienced a significant improvement in TIR due to a reduction in time spent in hyperglycemia > 250 mg/dl during daytime, as evidenced from FGM data. HbA1c declined only among patients with a baseline value > 7%, but marginally.

We elected time in hyperglycemia > 250 mg/dl during the day as the primary study endpoint, because we expected that, by reducing post-prandial glucose excursions, FIA would reduce spikes reaching such hyperglycemic range. Under this assumption, our finding is consistent with results of phase III RCTs on FIA, which showed an improvement in early post-meal glucose (18, 19). Our results are also similar to those reported by the GoBolus study (17), despite the patients cohort was younger and had a better baseline glucose control. In a larger population of patients with higher baseline HbA1c (8.1% [65 mmol/mol] versus 7.7% [60 mmol/mol] in our study), the GoBolus study showed a significant  – 0.2% ( – 2 mmol/mol) reduction in HbA1c and a reduction in time spent in hyperglycemia 180–250 mg/dl and > 250 mg/dl (17). It is well known that reduction in HbA1c is directly dependent from the baseline HbA1c value and it is easier to obtain greater (and more significant) HbA1c reductions when baseline HbA1c is higher (20). Indeed, we show that HbA1c declined significantly among participants with baseline HbA1c > 7.0% (53 mmol/mol), which was at least in part due to regression to the mean. Although average HbA1c was not significantly modified in all patients, TIR significantly improved by 2.7% (~40 min / day). According to available data, each 10% lower TIR (equal to 2.4 h / day) is associated with a 64% increase in the relative risk of retinopathy and 40% increase in the relative risk of microalbuminuria (14, 21). Indeed, TIR is emerging as a novel metric that complements HbA1c in assessing the overall impact of diabetes therapies on glycemic control (12). It should be noted that no clinically evident benefit could be observed among patients who had a baseline HbA1c value of 7% or lower and who were not using FGM.

Our study has typical limitations inherent to its retrospective design and real-world nature. The lack of a control group does not allow dissecting how much of the observed effect is due to the specific pharmacokinetics of FIA, or to random fluctuations, or to the change in therapy itself. In fact, any switch to a more modern therapy may elicit intrinsic improvements irrespectively of the true benefits of the new treatment. No benefit could be demonstrated for patients not wearing the FGM, for whom detailed data from self-monitoring of blood glucose (SMBG) were not available. In addition, we did not record how many correction boluses were the patients injecting at baseline and follow-up. Above all, we had no detailed information on the timing of the meals, on the timing of insulin injection with respect to the meal, and on meal composition. This prevented a formal evaluation of 1-h or 2-h post-prandial hyperglycemia. Though it is reasonable that glycemic spikes > 250 mg/dl are mainly due to the rapid post-prandial glucose surge, we acknowledge that the change in time spent with glucose value > 250 mg/dl may have other causes. The fact that glycemic improvement was more evident during daytime than during nighttime is consistent with a better coverage of post-prandial hyperglycemia. The trend reduction in hyperglycemia > 250 mg/dl during nighttime may be a consequence of a better control of post-dinner hyperglycemia. Even if our patients were always advised to inject bolus insulin before the meals (except when pre-prandial glucose is low), we cannot ensure that the changes we observe are due, at least in part, to the changes in the timing of bolus insulin injection. In other terms, similar results could be obtained by imposing an anticipation in the injection of other bolus insulins with respect to mealtime (22). Nonetheless, it is remarkable that, in free-living conditions and without any control over the timing of bolus injection, switch to FIA resulted in a significant reduction in the time spent with very high glucose values without changes in the time spent in hypoglycemia. Due to the purely retrospective nature of our study, we did not collect patient-reported outcomes, but we expect that limiting glycemic excursions could improve patients’ satisfaction with regards to treatment (17). In addition, FIA may grant a more flexible bolus administration, which can be an added value for young people with T1D.

It is also important to note that, in the absence of clinically meaningful improvements in HbA1c, the benefit of switching to FIA was observed only among T1D patients wearing the FGM system. Indeed, the widespread use of FGM, especially among people with T1D, today allows an unprecedented opportunity to analyze glucose trends and identify possible solutions. For example, a steep rise in glucose within the first 30–60 min after a meal can hardly be resolved by increasing bolus insulin dose, especially when the glucose values return to target range 2–3 h after the meal. In these cases, switch to FIA can yield a better synchronization of insulin action with post-prandial glucose appearance and reduce glycemic excursions. Consistently, we observed reductions in glycemic values > 250 mg/dl without any change in the dose of bolus insulin. On the other side, the significant increase in basal insulin dose, not accompanied by any change in the time spent in hypoglycemia, possibly reflects a more confident self-titration. Anyway, we acknowledge that such change was very small (< 1.0 IU/day) and unlikely to be clinically relevant. It should also be noted that we did not record information on the reasons driving the switch from prior bolus insulins to FIA, thereby making it impossible to rule out selection bias and assess generalizability of our finding. Finally, we only collected information at the first available follow-up visit after initiation of FIA and, therefore, cannot draw conclusions on whether and how long the observed improvements in glycemic control persist over time.

Notwithstanding such limitations, we believe our data provide a confirmation that the benefits of FIA demonstrated in RCTs can also be observed during the routine care of patients with T1D wearing a FGM system. This technology now enables enhanced opportunities of detecting glucose trends and applying the best solution for a patient-centric approach.

## Data Availability

Original data used in this manuscript are available from the corresponding author at a reasonable request.
